# Interleukin-37: associations of plasma levels and genetic variants in gout

**DOI:** 10.1186/s13075-023-03188-3

**Published:** 2023-10-18

**Authors:** Lucie Andres Cerezo, Adéla Navrátilová, Hana Hulejová, Markéta Pavlíková, Jakub Závada, Karel Pavelka, Ladislav Šenolt, Blanka Stiburkova

**Affiliations:** 1 Institute of Rheumatology, Na Slupi 4, 128 50 Prague 2, Czech Republic; 2 Department of Rheumatology, First Faculty of Medicine, Charles University, Prague, Czech Republic; 3 Department of Probability and Mathematical Statistics, Faculty of Mathematics and Physics, Charles University, Prague, Czech Republic; 4 Department of Pediatrics and Inherited Metabolic Disorders, First Faculty of Medicine, Charles University and General University Hospital in Prague, Prague, Czech Republic

**Keywords:** IL-37, Gout, Anti-inflammatory, IL-1 family, Gene polymorphism

## Abstract

**Objectives:**

IL-37 is an anti-inflammatory cytokine involved in inflammatory and autoimmune diseases. We aimed to investigate the association between IL-37 genetic variants, IL-37 plasma levels, and various clinical phases of gout.

**Methods:**

The study included a control group with no history of primary hyperuricemia/gout, (*n* = 50), asymptomatic hyperuricemia (*n* = 74), intercritical gout (*n* = 200), acute gouty flare (*n* = 18), and chronic tophaceous gout (*n* = 30). Plasma IL-37 was analysed using enzyme-linked immunosorbent assay. All coding regions and intron–exon boundaries of IL-37 and exons 1–5 were amplified and sequenced.

**Results:**

Plasma levels of IL-37 were significantly higher in asymptomatic hyperuricemic (*p* = 0.045), intercritical gout (*p* = 0.001), and chronic tophaceous gout (*p* = 0.021) cohorts when compared to control group. The levels of IL-37 in patients with acute gouty flare were comparable to control group (*p* = 0.061). We identified 15 genetic variants of IL-37: eight intron (rs2708959, rs2723170, rs2708958, rs2723169 rs2466448, rs3811045, rs3811048, rs2708944) and seven non-synonymous allelic variants (rs3811046, rs3811047, rs2708943, rs2723183, rs2723187, rs2708947, rs27231927), of which rs2708959 showed an over-presentation in gouty and acute flare cohorts (*p* = 0.003 and 0.033, respectively) compared to European population (minor allelic frequency MAF = 0.05) but not in control and hyperuricemic cohorts (p/MAF = 0.17/0.08 and 0.71/0.05, respectively).. On the contrary, rs3811045, rs3811046, rs3811047, and rs3811048 were underrepresented among individuals with tophaceous gout (MAF = 0.57) compared to European MAF 0.70–0.71, but not compared to the control cohort (MAF = 0.67).

**Conclusions:**

We demonstrated the up-regulation of IL-37 levels across the clinical phases of gout: asymptomatic hyperuricemia, intercritical, and chronic tophaceous gout compared to control. Moreover, 15 genetic variants of IL-37 were identified and their associations with the clinical variants of gout were evaluated.

## Introduction

Gout is a chronic disease with deposition of monosodium urate (MSU) crystals, resulting from increased urate concentrations. Patients with gout experience chronic recurring attacks accompanied by a variety of symptoms including inflammation, joint, and renal damage [1]. Hyperuricemia is a risk condition that predisposes a patient to the development of gout [2]. It is characterized by elevated plasma levels of uric acid, leading to MSU crystals formation or tophi deposition, which determines the severity of the symptoms. The genetic background plays an important role in the incidence of gout [1]. Although precise mechanisms have not been explored, the imbalance of the interleukin (IL)-1 cytokine family was thought to be one of the key processes underlying the gout pathology [3].

IL-37 is a unique member of the IL-1 family that functions as a natural suppressor of inflammation [3]. IL-37 is implicated in the pathogenesis of several autoimmune rheumatic diseases [4–7]; however, only few studies have focused on IL-37 in gout arthritis so far [8]. Initially, Zeng et al. demonstrated enhanced IL-37 expression in the peripheral blood mononuclear cells (PBMCs) of patients with gout, especially in the non-acute form [9]. Others showed elevation of serum IL-37 in gout patients, particularly in the active tophaceous gout and its association with the levels of C- reactive protein (CRP) and pro-inflammatory cytokines [10]. In vitro, IL-37 suppressed the production of inflammatory cytokines in THP-1 macrophages [9]. Moreover, IL-37 inhibited the immune reaction induced by MSU crystals in human and murine gout models [11]. Additionally, four rare genetic variants of IL-37 have been identified in gout patients, indicating a possible predisposition for the development of gout [12].

It is evident that IL-37 participates in the modulation of the immune response in gout arthritis [8]; however, little is known about the role of the IL-37 gene variation in the pathogenesis of gout. Therefore, we aimed to investigate the link between plasma levels of IL-37 and genetic variants of IL-37 in relation to the development and progression of gout.

## Materials and methods

The study cohort included a control group of 50 subjects with no history of primary hyperuricemia/gout, 74 patients with asymptomatic hyperuricemia, 200 patients with intercritical gout, 30 patients with chronic tophaceous gout, and 18 patients with acute gouty flares. Gout patients met the 1977 American Rheumatism Association preliminary classification criteria for acute arthritis of primary gout [13]. Asymptomatic hyperuricemic patients were classified as having serum uric acid (SUA) > 420 μmol/L for men and SUA > 360 μmol/L for women. Patients with secondary gout and other purine metabolic disorders associated with pathological concentrations of SUA were excluded. High-performance liquid chromatography determination of hypoxanthine and xanthine in urine was performed on an Alliance 2695 and a 2998 photodiode array detector (Waters, Milford, MA, USA) as previously described [14]. The analysed cohorts were selected based on uric acid levels from a previously reported set of 250 hyperuricemia/gout patients and 132 control subjects with no history of primary hyperuricemia, gout, or autoimmune disease [15, 16] with descending levels of serum urate. All included subjects were aged 18 years and more. Written informed consent was obtained from each subject, and all tests were performed according to standards set by the institutional ethics committee (project no. 6181/2015). All procedures were carried out in accordance with the Declaration of Helsinki. All demographic, biochemical, and medical treatment data are presented in Table 1.

**Table 1:** Main demographic, biochemical, and genetic characteristics of the control (*N* = 50), asymptomatic hyperuricemic (*N* = 74), intercritical gout (*N* = 200), acute gouty flare (*n* = 18), and chronic tophaceous gout (*N* = 30) cohorts

	**Control subjects (*N* = 50)**		**Asymptomatic hyperuricemia patients (*N* = 74)**		**Intercritical gout patients (*N* = 200)**		**Acute gouty flare patients (*N* = 18)**		**Chronic tophaceous gout patients (*N* = 30)**		**Fisher’s test** ***p*-value**
	***N***	**%**	***N***	**%**	***N***	**%**	***N***	**%**	***N***	**%**	
Sex M/F	12/38	24.0/76.0	54/20	73.0/27.0	179/21	89.5/10.5	18/0	100.0/0.0	28/2	93.3/6.7	< 0.0001
Familial occurrence	Not collected		26	40.0	79	41.1	9	50.0	12	42.9	0.8820
No treatment	50	100.0	27	41.5	16	8.6	3	18.8	6	21.4	0.0005
Allopurinol treatment	Not applicable		38	58.5	152	81.3	11	68.8	18	64.3	
Febuxostat treatment			0	0.0	19	10.2	2	12.5	4	14.3	
*p*.Q141K, MAF	8	8.2	27	18.2	96	24.0	6	16.7	14	23.3	0.0055
IL-37 data available	50	100.00	63	85.1	176	88.0	18	100.00	25	83.33	< 0.0001
	**Median (IQR)**	**Range**	**Median (IQR)**	**Range**	**Median (IQR)**	**Range**	**Median (IQR)**	**Range**	**Median (IQR)**	**Range**	**Kruskal–Wallis test** ***p*-value**
Age of onset, years	Not applicable		35.0 (31.5)	11–76	40.0 (21.0)	12–84	38.0 (18.0)	18–61	36.0 (21.0)	9–68	0.3236
Current age, years	46.5 (31.2)	24–78	43.5 (33.2)	19–78	50.0 (22.0)	19–90	47.0 (11.2)	35–63	49.0 (23.2)	29–73	0.1053
BMI (*N* = 46/66/179/17/26)^c^	25.6 (5.5)	17.9–38.5	29.0 (6.3)	21–43.6	28.4 (5.9)	19.3–50	28.1 (4.6)	23.4–32.8	28.2 (5.0)	20.7–38.6	0.0001
SUA without treatment, μmol/l (*N* = 50/54/140/12/22)^c^	329.0 (117.2)	208–544	458.5 (98.5)	371–635	462.0 (115.8)	181–685	504.0 (105.5)	406–616	461.5 (161.0)	245–647	< 0.0001
SUA with treatment, μmol/l (*N* = 0/40/170/14/23)^c^	Not applicable		415.5 (144.5)	207–628	360.5 (121.8)	131–725	430.5 (158.8)	238–579	438.0 (125.5)	167–808	0.0017
FE-UA^a^ (*N* = 0/68/196/17/29)	Not measured		3.8 (1.9)	1.7–13.2	3.7 (1.6)	0.8–11.8	3.2 (1.1)	2.1–5.4	4.5 (3.0)	1.2–13.1	0.3099
GFR-MDRD, ml/min^a^ (*N* = 50/45/133/11/17)	79.0 (20.2)	45–122	86.0 (25.0)	46–138	86.0 (26.0)	24–132	92.0 (13.1)	31–117	81.0 (30.5)	27.5–130.5	0.0864
Serum creatinine, μmol/l^a^ (*N* = 50/70/198/18/29)	71.5 (17.8)	56–105	79.0 (19.5)	44–132	80.8 (17.4)	48–226	82.0 (21.0)	64–189	91.5 (20.5)	57–179	0.0003
Max CRP^b^ (*N* = 50/66/180/18/25)^c^	1.3 (2.2)	0.2–10	2.6 (4.2)	0.1–153.1	3.7 (5.7)	0.2–224.4	4.8 (16.8)	0.5–194.7	9.2 (31.8)	0.2–268	< 0.0001

*BMI* body mass index, *CRP* C-reactive protein, *FE-UA* fractional excretion of uric acid, *GFR* glomerular filtration rate, *IQR* interquartile range, *MDRD* modification of diet in renal disease

^a^Mean of measurements taken during the follow-up

^b^Maximum of measurements taken during the follow-up

^c^There were missing data for some parameters; in case missing data amounted to 5% or more, the real *N* is mentioned in parentheses

Plasma samples were collected and stored at – 80 °C until analysis. The levels of C-reactive protein were assessed by a turbidimetric technique using an Olympus Biochemical Analyser (Olympus CO Ltd., Tokyo, Japan). IL-37 in plasma was analysed by the human enzyme-linked immunosorbent assay (ELISA) kit (Adipogen Life Sciences, Liestal, Switzerland).

Genomic DNA was extracted from EDTA-treated whole blood using a Exgene™ Clinic SV (Gene All, Germany). All five protein-coding exons were amplified using PCR and purified using a PCR DNA Fragments Extraction Kit (Geneaid, New Taipei City, Taiwan). DNA sequencing was performed with a DNA sequencer (Applied Biosystems 3130 Genetic Analyzer; Thermo Fisher Scientific, Waltham, MA, USA). Primer sequences and PCR conditions are available upon request. The reference sequence was defined as version ENST00000263326.

In the pilot analysis, 50 control subjects, 50 subjects with asymptomatic hyperuricemia, 50 patients with intercritical gout, 17 patients with acute gouty flares, and 28 patients with chronic tophaceous gout were genotyped. Variant allele frequencies in the above-mentioned groups were compared with the European MAF (EXAC database) using the binomial test. Variants with statistically significant allele frequency differences compared to the European population (*p* < 0.05) were genotyped in an expanded cohort of 74 hyperuricemic patients, 200 gout patients, 18 patients with gouty flares, and 30 patients with chronic tophaceous gout.

Variant allele frequencies were compared with the European MAF using the binomial test and with the control group using Fisher’s exact test. Differences in IL-37 levels between control/hyperuricemic/gout groups were compared pairwise, using the two-sample Wilcoxon test with the Benjamini–Hochberg correction for multiple testing. Spearman’s correlation was used to correlate levels of IL-37 and CRP. *p* values < 0.05 were considered significant. All analyses were performed using the statistical language and environment R, version 4.0.2.

## Results

**Fig. 1:**
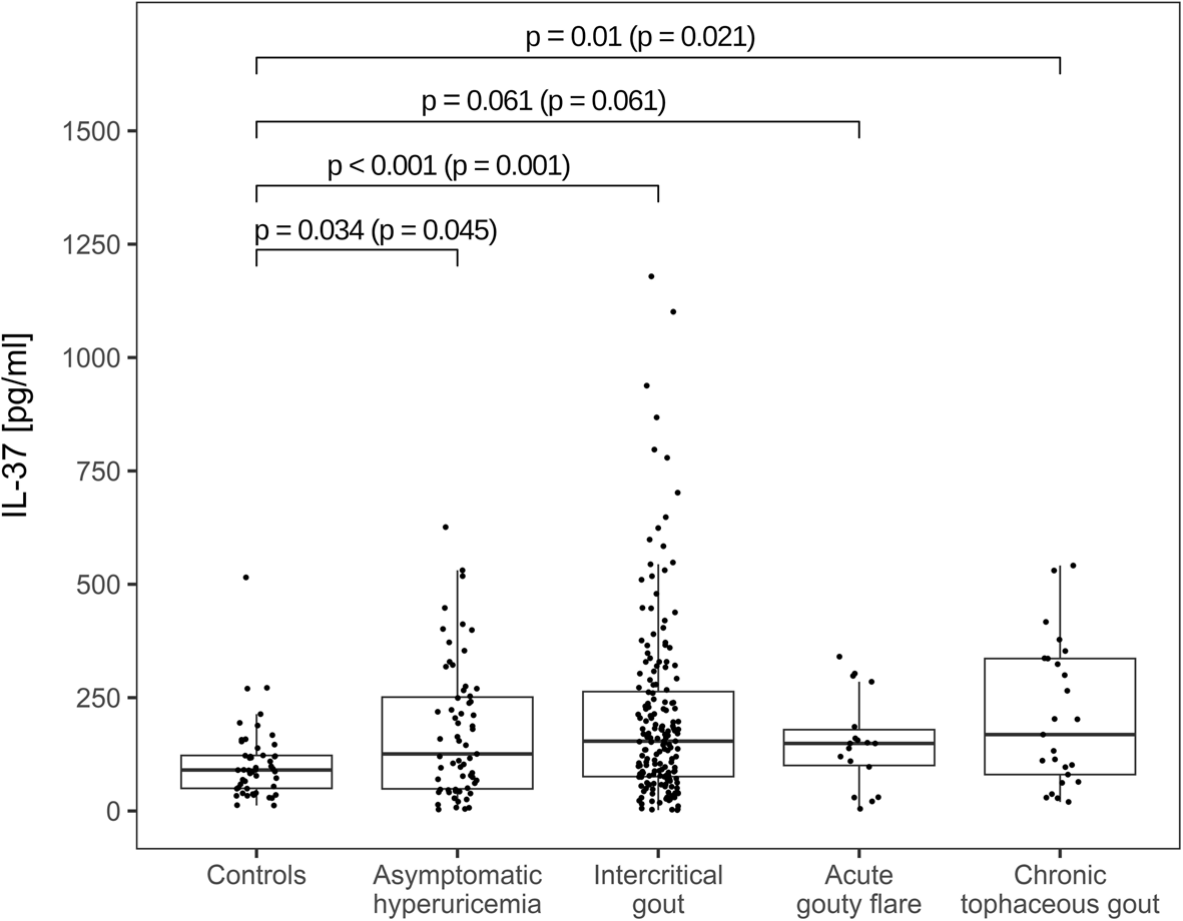
Plasma levels of IL-37 in controls, asymptomatic hyperuricemia, intercritical gout, acute gouty flare and chronic tophaceous gout patients. Two-sample Wilcoxon test *p*-values, unadjusted (with Benjamini–Hochberg adjustment for multiple comparisons)

Plasma levels of IL-37 were significantly higher in asymptomatic hyperuricemic (*p* = 0.045), intercritical gout (*p* = 0.001), and chronic tophaceous gout (*p* = 0.021) cohorts when compared to control group. The levels of IL-37 in patients with acute gouty flare were not significantly different from the control group (*p* = 0.061) (Fig. 1), both without and with Benjamini–Hochberg correction for multiple comparisons.

Of the fifteen variants, only rs2708959 showed a significant difference in the variant allele frequency between the gout/gout flare cohorts and the European population MAF (Table 2). Compared to European MAF = 0.05, we found an allele frequency 0.085 in gout and 0.139 in the gout flare cohorts (*p* = 0.003 and 0.033, respectively). There was no statistically significant difference from European MAF for the control and asymptomatic hyperuricemic cohorts (p/MAF = 0.17/0.08 and 0.71/0.05, respectively). We have also found that rs3811045, rs3811046, rs3811047, and rs3811048 were underrepresented among individuals with tophaceous gout, with MAF = 0.57 for all four compared to European MAF 0.705, 0.705, 0.703, and 0.704 respectively. In no variants and no cohorts, allele frequencies varied from the frequency in the normouricemic cohort (Table 2).

**Table 2:** Number and frequency of detected variant alleles and levels of IL-37 in control subjects (*N* = 50), asymptomatic hyperuricemic (*N* = 74), intercritical gout (*N* = 200), acute gouty flare (*N* = 18), and chronic tophaceous gout (*N* = 30) cohorts

	IL-37 [pg/ml]	**rs2708959**	**rs2723170**	**rs2708958**	**rs2723169**	**rs2466448**	**rs3811045**	**rs3811046**	**rs3811047**	**rs3811048**	**rs2708943**	**rs2723183**	**rs2708944**	**rs2723187**	**rs2708947**	**rs27231927**
	Median (IQR)	***n*** **MAF**	***n*** **MAF**	***n*** **MAF**	***n*** **MAF**	***n*** **MAF**	***n*** **MAF**	***n*** **MAF**	***n*** **MAF**	***n*** **MAF**	***n*** **MAF**	***n*** **MAF**	***n*** **MAF**	***n*** **MAF**	***n*** **MAF**	***n*** **MAF**
European population (NCBI)		0.050	0.080	0.081	0.079	0.079	0.705	0.705	0.703	0.704	0.081	0.082	0.079	0.081	0.082	0.082
Control subjects (*N* = 50/50^a^)	90.4 (49.9–122.5)	8	8	8	8	8	67	67	67	67	8	8	8	10	7	7
		0.080	0.080	0.080	0.080	0.080	0.670	0.670	0.670	0.670	0.080	0.080	0.080	0.100	0.070	0.070
Asymptomatic hyperuricemic (*N* = 111/146^a^)	158.9 (68.8–318.1)	8	8	8	8	8	109	109	109	109	10	10	9	8	8	8
		0.054	0.054	0.054	0.054	0.054	0.736	0.736	0.736	0.736	0.068	0.068	0.061	0.054	0.054	0.054
Intercritical gout (*N* = 182/206^a^)	154.0 (76.3–270.8)	34	34	34	34	36	285	284	284	284	33	34	34	40	40	40
		**0.085**	0.085	0.085	0.085	0.090	0.713	0.710	0.710	0.710	0.083	0.085	0.085	0.100	0.100	0.100
Chronic tophaceous gout (*N* = 20/30^a^)	149.1 (83.7–188.5)	**6**	**6**	**6**	**6**	**6**	**25**	**25**	**25**	**25**	**4**	**3**	**4**	**4**	**3**	**3**
		**0.139**	0.139	0.139	0.139	0.139	0.639	0.639	0.639	0.639	0.083	0.056	0.083	0.083	0.083	0.083
Acute gouty flare (*N* = 25/20^a^)	168.5 (80.3–336.2)	*5*	5	5	5	5	34	34	34	34	2^b^	2^b^	2^b^	3	3	3
		0.083	0.083	0.083	0.083	0.083	**0.567**	**0.567**	**0.567**	**0.567**	0.034	0.034	0.034	0.050	0.050	0.050

The cases where binomial test (compared to the European population MAF) *p*-value < 0.05 are in bold

*IQR* interquartile range, *MAF* minor allelic frequency, *n* number of alleles found

^a^Number of individuals with measured IL-37 levels/genotyped

^b^For rs2708943, rs2723183, and rs2708944, only 29 out of 30 individuals with acute gouty flare were genotyped

## Discussion

IL-37 is a key suppressor of innate immunity and a master regulator of inflammation at mucosal surfaces. Here, we show that IL-37 is significantly elevated in the plasma of patients with intercritical and tophaceous gout. This evidence supports the implication of IL-37 in the pathogenesis of gout. In the last decade, several research groups have focused on IL-37 and its association with gout [9–11]. To the best of our knowledge, we are the first to demonstrate an elevation of IL-37 in the plasma of tophaceous gout Caucasian patients. Our findings are congruent with the data of Ding et al. in Chinese gout patients [10] and imply an association of IL-37 with the deposition of MSU crystals. This is further supported by the accumulation of IL-37 in the synovium surrounding the tophus [11] and by in vitro studies using MSU crystals [3, 9, 11]. In contrast to others [10], we found significant differences in the levels of IL-37 in hyperuricemic compared to control subjects. These adverse results may be caused by the fact that our cohort was about five times larger in contrast to the abovementioned study [10]. Moreover, IL-37 was not elevated in the plasma of acute gouty patients compared to controls, which is inconsistent with previous study [10]. This finding is surprising as acute gout is an inflammatory reaction to the deposition of MSU crystals. As our study involved only 20 patients with acute gouty flare, further analysis on a larger cohort is needed to confirm this result. In contrast to Ding et al. who showed an association of IL-37 with clinical inflammatory markers and cytokines in patients with gout [10], we found no correlation between IL-37 and CRP levels. It is well established that anti-rheumatic therapy can suppress inflammation and modify cytokine levels. The study by Ding et al. [10] did not include data on the therapeutic intervention, which makes the comparison with our results rather problematic. Finally, it must be noted that the data on circulating IL-37 vary across the published studies (our study included), and the levels of IL-37 also differ by an order of magnitude [9–11]. One reason for the high variability in circulating IL-37 levels is the use of different ELISA kits, as shown in a recently published study [17].

However, no published study has analysed IL-37 levels across the four clinical phases of gout: asymptomatic hyperuricemia, intercritical gout, gouty flares, and chronic tophaceous gout. In our study, we did not find any significant associations of common or rare IL-37 variants with the abovementioned groups; however, we demonstrated a significant underrepresentation of common variants whose functional impact on IL-37 expression or stability is currently unknown.

Clinical studies have demonstrated that IL-37 gene variant rs3811047 G > A is associated with susceptibility to rheumatoid arthritis [18] and ankylosing spondylitis [19, 20]. Notably, a recent study by Kluck et al. [12] showed a devastating impact of four rare IL-37 variants (p.A144P, p.G174Dfs*16, p.C181*, and p.N182S) on protein functions in the pathogenesis of gout. However, in our study, we have not identified any rare variants in *IL-37* gene. The frequencies of minor alleles for each SNP were compared to published frequencies in dbSNP for cohorts with similar ethnic backgrounds to our subjects, i.e. majority European descent. Of the fifteen identified variants, only rs2708959 showed a significant difference in the variant allele frequency between the hyperuricemic/gout/gout flare cohorts and the European population MAF. Compared to European MAF = 0.05, we found a- significant allele frequency 0.085 in gout and 0.150 in the gout flare cohorts. However, this variant is located in upstream intronic region, and currently, there are no analyses available regarding its impact on IL-37 protein or its association with phenotype. We have also found that rs3811045, rs3811046, rs3811047, and rs3811048 were underrepresented among individuals with tophaceous gout, with MAF = 0.57 for all four compared to European MAF 0.705, 0.705, 0.703, and 0.704, respectively. Upstream intronic variants rs3811045 and rs3811046 have not been investigated in rheumatic diseases; however, a significant association of rs3811045 with an increased risk of COVID-19 [21] and an association of rs3811046 with susceptibility to pulmonary tuberculosis [22] were recently demonstrated.

Non-synonymous allelic variants 3811046 (p.Gly31Glu, p.Gly31Ala, and/or p.Gly31Val) and 3811047 (p.Thr42Ala, p.Thr42Ser) located in exon 2 are missense variants with reported limit observations. No associations of rs3811046 were found in rheumatoid arthritis [23], open-angle glaucoma [24], and tuberculosis [25]. However, three significant associations were reported in periodontal inflammation [26], COVID-19 [21], and as a decreased genetic risk factor in Graves’ disease [27].

Variant rs3811047 has been associated with susceptibility to or protection against several infectious, inflammatory, and autoimmune diseases as ankylosing spondylitis, rheumatoid arthritis, auto-immune thyroid disease, Behcet’s disease, and Vogt-Koyanagi-Harada disease [28]. The current study showed that TG genotype of rs3811046 may increase the risk of developing COVID-19 [21].

This finding may be influenced by study limitations—first, although our sample size of 372 hyperuricemia/gout subjects is large for studies involving deep physiological phenotyping of hyperuricemia/gout, it is quite small for genetic studies, which often include hundreds to thousands of subjects—some functional variants may have gone undetected. Second, we studied genetic variants in transcribed regions and exon–intron boundaries only, and therefore, genetic variants outside these regions would have gone undetected.

The “Common Disease, Multiple Common and Rare Variant” model argues that genetic susceptibility to common diseases such as gout does not reside only in common genetic variants but rather in a multiplicity of individually rare variants with high penetrance. This theory has been fully confirmed by the relationship between hyperuricemia, gout, and functional variants in the major uric acid transporter ABCG (ATP-binding cassette subfamily G member) [15]. Our results suggest that not only rare variants but also reduced presentation of common variants in the IL-37 gene may influence disease progression. Therefore, haplotype analysis is a useful approach to understanding the genetic association between IL37 gene SNPs and diseases, but due to the limited data available, further studies and experimental verifications are necessary.

## Conclusion

Taken all together, we showed an elevation of serum IL-37 across the clinical phases of gout compared to controls. Furthermore, the detection of 15 genetic variants of IL-37 and their association with the clinical phases of gout indicates that underrepresentation of common variants in the IL-37 gene may potentially influence progression of gout.

## Data Availability

The datasets used and/or analysed during the study are available from the corresponding author on reasonable request.
